# Menopause experiences among British South Asian women: An exploratory qualitative study

**DOI:** 10.1177/17455057261478338

**Published:** 2026-08-11

**Authors:** Shahara Aziz Ellahi, Vikram Talaulikar, Jessica Wibisono, Aimee Spector

**Affiliations:** 1 4919 Department of Clinical, Educational and Health Psychology, University College London, London, United Kingdom; 2 Department of Reproductive Medicine, University College London Hospitals NHS Foundation Trust, London, United Kingdom

**Keywords:** menopause, British South Asian women, thematic analysis, barriers, ethnic minorities

## Abstract

**Background:**

While menopause is a universal biological transition, individual experiences and engagement with healthcare vary considerably and are significantly shaped by intersecting cultural, social, and relational contexts.

**Objective:**

To explore how British South Asian women experience and understand menopause, and how cultural and healthcare contexts influence symptom recognition, coping, and engagement with care.

**Design:**

Qualitative interview study using semi-structured interviews conducted online.

**Methods:**

A reflexive thematic analysis approach was used to analyse data collected from interviews. The analysis captured women’s accounts of menopausal symptoms, understanding of menopause, coping strategies, healthcare experiences, and perceived support needs.

**Results:**

Fifteen British South Asian women aged 43–61 years across peri- and postmenopausal stages participated. Two overarching domains were identified: (1) experiences and understanding of menopause, and (2) coping, support, and access to information. Limited menopause literacy, cultural silence, and stigma contributed to delayed recognition of symptoms and uncertainty about whether changes were menopausal. Women described a multidimensional symptom burden affecting physical, emotional, and social functioning. Healthcare encounters strongly influenced engagement with care, with dismissive consultations discouraging help-seeking and validating interactions enhancing understanding, sense of control, and openness to treatment options, including hormone therapy. In the context of limited formal support, women relied on self-management strategies, faith, and informal peer networks. Limited understanding among male partners was commonly reported, although generational shifts toward greater openness were evident.

**Conclusions:**

British South Asian women’s menopause experiences are shaped by intersecting cultural, relational, and healthcare factors. Improving culturally sensitive communication, menopause literacy, and inclusive, community-based support may help reduce inequities in menopause care and enhance engagement with services.

## Introduction

Menopause is a significant life transition characterised by fluctuating and declining ovarian hormone levels alongside the cessation of menstruation. Although commonly associated with vasomotor symptoms such as hot flushes and night sweats, menopause also encompasses a broad range of psychological, cognitive, and physical symptoms including sleep disturbance, anxiety, low mood, musculoskeletal pain, fatigue, and difficulties with concentration.^1,2^ For some women, these symptoms can substantially affect emotional wellbeing, occupational functioning, relationships, and quality of life.^3^ However, experiences of menopause are not uniform and are shaped by broader social, cultural, and relational contexts.

Increasing evidence highlights important ethnic and cultural differences in menopause experiences, symptom appraisal, and healthcare engagement. Research suggests women from ethnic minority and socioeconomically disadvantaged backgrounds may experience earlier menopause onset, more complex symptom profiles, and inequitable access to menopause support and treatment.^4,5^ Studies involving South Asian populations have reported greater prevalence of musculoskeletal symptoms and mood disturbances, alongside lower awareness of menopausal symptoms compared to White British populations.^6^ Earlier menopause onset has also been reported among some South Asian populations, which may contribute to delayed symptom recognition and unmet support needs among women in their forties.^7^

Despite growing public discourse surrounding menopause in the UK, disparities in access to menopause care and hormone therapy (HT) persist. Recent UK qualitative research has shown that Black and Asian women report lower rates of HT use, dissatisfaction with menopause consultations, concerns surrounding stereotyping, and mistrust toward healthcare systems.^8^ Women from ethnic minority backgrounds have also described menopause as occurring alongside significant caregiving responsibilities, employment pressures, and broader social inequalities, which shape experiences of symptom management and help-seeking. More broadly, ethnic minority communities continue to experience structural healthcare inequalities and culturally insensitive care across a range of health conditions, which may reinforce mistrust and disengagement from healthcare services.^9,10^

These disparities occur within wider sociocultural contexts that shape women’s understandings of menopause and help-seeking behaviours. Within some South Asian communities, reproductive and sexual health topics may remain highly private or stigmatised, contributing to limited intergenerational discussion and reduced menopause literacy.^11^ This may reflect broader cultural norms within some South Asian communities in which discussions of women’s reproductive health are considered inappropriate or uncomfortable, with menopause often carrying greater stigma than menstruation and consequently remaining surrounded by silence and secrecy.^12^

Previous research has shown that menopause is often normalised as a private aspect of ageing rather than understood as a health transition requiring support or intervention.^13^ Limited culturally relevant information, language barriers, and experiences of culturally insensitive care may further discourage women from seeking formal healthcare support.^10^ Menopause may therefore be experienced within intersecting cultural, relational, and healthcare systems that shape how symptoms are recognised, interpreted, and managed.

Menopause experiences are increasingly understood as socially and relationally embedded rather than solely biological events. Hunter and Smith highlight that menopause may involve wider changes in identity, social roles, relationships, and cultural expectations.^3^ The Common-Sense Model (CSM) of self-regulation provides a useful framework for understanding these processes, proposing individuals develop cognitive and emotional representations of health conditions which subsequently shape coping responses and help-seeking behaviours.^14^ Applying the CSM within the context of menopause may therefore help explore how cultural beliefs, social norms, and healthcare experiences influence women’s interpretations and management of menopausal symptoms.

Despite increasing recognition of menopause within UK public health discourse, qualitative research exploring the experiences of British South Asian women remains limited. Existing studies have frequently aggregated ethnic minority groups together or focused on international populations, limiting understanding of the culturally specific experiences of South Asian women living in the UK.^10,15^ In particular, little is currently known about how British South Asian women recognise and interpret menopausal symptoms, navigate healthcare encounters, access support, and negotiate menopause within wider family and cultural systems.

This study therefore aimed to explore how British South Asian women experience, interpret, and manage menopause, and how cultural and contextual factors shape symptom appraisal, coping, and healthcare engagement. Specifically, the study explored: (1) how menopausal symptoms were recognised and understood; (2) attitudes and beliefs surrounding menopause; (3) experiences of emotional wellbeing throughout the menopausal transition; and (4) coping strategies, support systems, and healthcare experiences among British South Asian women.

## Methods

### Study design

Qualitative semi-structured interview study using reflexive thematic analysis.

### Ethical considerations

Ethical approval for this study was granted by the University College London (UCL) Research Ethics Committee (Ethics Approval Number: 28691/001).Prior to data collection, all participants were provided with an information sheet via email and completed an electronic written informed consent form via the Qualtrics platform. Participants were informed of their right to withdraw at any stage, and all data were fully anonymised to protect identity.

### Participants and recruitment

Fifteen British South Asian women aged 43–61 years were recruited across England using purposive sampling. Recruitment materials were circulated via social media and community and faith-based organisations. Community organisations such as Langley Islamic Centre, Hounslow Sister Circle and Burslem Mosque circulated the study advertisement through existing networks; they did not identify, screen, or directly approach potential participants. The advertisement was additionally forwarded to community WhatsApp groups and platforms by the researcher and members of these communities.

The inclusion criteria required participants to: (1) self-identify as Bangladeshi, Indian, Pakistani, or Sri Lankan; (2) be English-speaking; (3) be resident in England; and (4) be currently experiencing peri- or post-menopause, determined using STRAW+ criteria.^16^ The exclusion criteria omitted women with severe mental illness, disabling medical conditions, or surgically induced menopause, for example following bilateral oophorectomy.

### Data collection

Following recruitment, participants were contacted by email with additional information and an opportunity to ask further questions.

Individual semi-structured interviews were conducted online using Microsoft Teams or Zoom, which lasted 40–60 minutes. The interview schedule was exploratory and covered four domains: experiences of menopause and its physical and emotional impact; cultural beliefs and knowledge about menopause; sources of support and engagement with healthcare; and barriers and facilitators to help-seeking (See Supplementary Material A).

Data collection was conducted over a duration of 7 months, commencing in January 2025 and concluding in July 2025. Sampling and interviews continued until sufficient conceptual depth and richness of data were achieved, with no substantially new concepts emerging in later interviews.

Participants received a £10 voucher in recognition of their time and were offered a summary of the study findings.

### Data analysis

Interviews were audio-recorded and transcribed verbatim. Data were analysed using reflexive thematic analysis following Braun and Clarke’s framework.^17^ Initial inductive coding was conducted across transcripts, with related codes iteratively clustered into candidate themes and organised into domains.

To further ensure the quality and validity of the thematic analysis, the research process was guided by Yardley’s four principles for evaluating qualitative research: sensitivity to context, commitment and rigour, transparency and coherence, and impact and importance.^18^ Sensitivity to context and commitment were demonstrated through the purposive recruitment of a culturally specific sample from trusted community spaces and the depth of the semi-structured interviews.

Transparency and coherence were maintained through independent double-coding of 20% of the transcripts to enhance analytic rigour. Initial coding and theme development were undertaken by SAE, with a second researcher (JW) independently reviewing a subset of transcripts. Coding decisions and theme development were subsequently discussed with the wider research team to refine and finalise the themes. A reflective journal was used throughout to monitor and evaluate researcher positionality.

NVivo software (version 14.2.4.3) was used to organise and manage the data. The study was conducted and reported in accordance with the Consolidated Criteria for Reporting Qualitative Research (COREQ) checklist.^19^

### Researcher reflexivity

Interviews were conducted by trainee clinical psychologist SAE. SAE’s position and identity as a British South Asian female, had an inevitable role in shaping the research and data collection. Shared cultural background facilitated rapport and trust with participants, however this proximity risked assumptions of shared understanding thus obscuring important contextual details. To address this, reflexivity was embedded across the process. The use of a reflective journal to document emerging assumptions, decisions, and developing interpretations allowed a critical examination of how cultural background, professional training, and personal perspectives might influence the data collection and analysis.

## Results

### Participant characteristics

A total of 15 women participants (43-61 years) who were in perimenopause (n = 4), late perimenopause (n = 4), and post-menopause (n = 7) were interviewed. A majority of the sample had never used hormone therapy (HT) (n=11), while a smaller number reported current or previous use. The sample was composed of Pakistani (n = 6), Indian (n = 5), Bangladeshi (n = 2), or Sri Lankan (n = 2) women (Tables 1 and 2).

**Table 1.** Participant characteristics.

Participant characteristics	
Characteristics	N
Age	
40-45	2
46-50	4
50-55	4
56-60	5
Hormone Therapy (HT) Status	
Current User	3
Previous User	1
Never Used	11
Menopausal Stage	
Early Perimenopausal	4
Late Perimenopausal	4
Postmenopausal	7
Ethnic Origin	
Bangladeshi	2
Pakistani	6
Sri Lankan	2
Indian	5
Education	
Secondary school (GCSE or equivalent)	1
Bachelor’s Degree	10
Postgraduate degree (Master’s/Professional/Doctoral)	3
Prefer not to say	1

**Table 2.** Thematic analysis of participant experiences.

Domain	Theme
1. Understanding and experiencing menopause	1.1 Silence, stigma, and taboo
	1.2 Physical and Emotional Upheaval
	1.3 Navigating Roles and Relational Support
	1.4 Generational Shift
	1.5 Encounters with healthcare: dismissal and validation
2. Coping, seeking support, and accessing information	2.1 Coping, resilience, and strategies of control
	2.2 Knowledge, education, and the power of information
	2.3 Male Understanding and Engagement

Thematic analysis generated eight sub themes across two overarching domains: (1) Experiences and understanding of menopause and (2) Coping, support, and access to information.

To demonstrate the complete depth, context, and semantic richness of the dataset, an expanded matrix of supporting participant quotations across all primary domains and sub-themes is provided in Supplementary Material B. All participant names used in quotations are pseudonyms.

### Domain 1: Experiences and understanding of menopause

#### Silence, stigma, and delayed recognition

For many participants, menopause was described as a culturally silenced and stigmatised experience, shaped by broader norms surrounding privacy and modesty in discussions of women’s reproductive health. Women frequently reflected on growing up in family and community environments where topics such as menstruation, ageing, and menopause were rarely discussed openly, particularly between generations of women. As a result, many entered menopause with little prior understanding of what to expect, contributing to uncertainty and limited preparedness when symptoms emerged.

“Topics about menopause, it’s really kept secret. It’s not really announced. It’s not really publicized.” (Priya, 43, Early Perimenopausal)

For several women, this silence was experienced as both cultural and relational, reinforced through the absence of conversations within families and communities. Participants described limited opportunities to learn about menopause from older female relatives, often resulting in feelings of uncertainty regarding who they could speak to or seek reassurance from.

“I think in Asian culture you don’t talk about this kind of thing. And that’s quite hard because you don’t know who you talk to.” (Zara, 49, Postmenopausal)

This lack of open dialogue shaped not only women’s knowledge of menopause, but also how symptoms were recognised and interpreted. Participants frequently described uncertainty regarding whether emotional and physical changes were attributable to menopause or other life circumstances, often struggling to contextualise symptoms in the absence of prior awareness or discussion. Several women reflected emotional difficulties were endured privately, with limited language or permission to openly express distress.

“Some women out there who don’t even know that they’re going through it but they’re dealing with these symptoms and haven’t got a clue …you have these emotions, and you have these feelings that you’re not allowed to, you’ve got to suppress them because you’re not allowed to talk about them.” (Riya, 46, Early Perimenopausal)

Beyond delayed recognition, participants described emotional consequences associated with this silence, including shame, loneliness, and self-doubt. Several women reflected on navigating symptoms privately while maintaining family and caregiving responsibilities, often feeling menopause should be managed quietly rather than openly acknowledged or supported. For some, menopause was framed as something deeply personal or even shameful, reinforcing feelings of invisibility.

“I’ve never ever sat in a space with middle-aged women, women who are older than me even, who have ever really spoke about it, it’s this shameful thing.” (Fatima, 58, Postmenopausal)

Women also described a prevailing expectation to simply “get on with it,” reflecting both resilience and a perceived cultural norm of enduring discomfort privately. However, some participants observed signs of generational change, describing greater openness among peers and younger women, who appeared increasingly willing to discuss menopause and seek information.

Collectively, these accounts suggest cultural silence shaped not only menopause literacy, but women’s ability to recognise, legitimise, and seek support for menopausal changes. Rather than menopause being understood as an expected life transition, many participants described navigating symptoms in uncertainty, often without a clear framework through which to understand their experiences.

#### Physical and emotional disruption

Participants described menopause as a multidimensional and disruptive experience, characterised by interconnected physical, emotional, and cognitive changes. Women reported a broad range of symptoms including hot flushes, sleep disturbance, musculoskeletal pain, vaginal dryness, bloating, mood changes, anxiety, forgetfulness, and “brain fog”. Symptoms were often experienced simultaneously, making menopause difficult to recognise and interpret, particularly alongside other midlife stressors.

“I kept thinking, is this the menopause or is it just the stress of my divorce? Everything was happening at once, I couldn’t separate it.” (Tania, 52, Postmenopausal)

Several participants described symptoms as overwhelming and difficult to manage, emphasising how physical discomfort frequently intersected with emotional distress and cognitive difficulties.

“Tinnitus, sweats, broken sleep, mood swings, frozen shoulder, cloudy brain, difficult to recall, bloating and gas, itchy skin.” (Neha, 48, Early Perimenopausal)

“There were the sweats, discomfort during the night, difficulty sleeping, this hotness in your system, but also this loss of sort of this low mood, depression, anxiety.” (Priya, 43, Early Perimenopausal)

Beyond physical symptoms, women frequently described menopause as disrupting their emotional wellbeing, confidence, and sense of self. Several participants reflected on feeling unlike themselves, describing emotional changes felt unfamiliar and difficult to explain to others.

“You feel like a different person…like something’s being taken away from you. So, I feel like I’m not the person that I used to be.” (Simi, 47, Early Perimenopausal)

For some, emotional symptoms significantly affected confidence and everyday functioning.

“I was so nervous. I was driving home…and I just stopped the car on the motorway and I was like, I can’t do anymore…I totally lost my confidence.” (Bushra, 51, Postmenopausal)

Taken together, these accounts suggest menopause was experienced not simply as a set of physical symptoms, but as a disruptive transition affecting emotional wellbeing, functioning, and identity, often occurring alongside broader relational and life stressors.

#### Roles, relationships, and generational change

Participants frequently described menopause as occurring alongside multiple relational and caregiving responsibilities, including employment, caring for children or ageing parents, and maintaining family roles. Several women reflected on the pressure to continue fulfilling these responsibilities despite significant physical and emotional changes, often feeling their own wellbeing became secondary to the needs of others.

“I feel a whole lot of reliance doing certain things…so it’s really affecting my family especially.” (Priya, 43, Early Perimenopausal)

Many participants described an expectation to continue coping privately, with menopause often framed as something women were expected to endure rather than openly acknowledge. This frequently reinforced feelings of emotional isolation and invisibility within family environments.

“…your kind of just going through life on your own, expected to get on with it and cope as best as you can.” (Atifa, 58, Postmenopausal)

Support from partners and family members varied considerably. While some women described limited understanding from older relatives, others reflected on supportive relationships that helped normalise their experiences and reduced feelings of loneliness. Generational differences emerged prominently across accounts. Older generations of women were frequently perceived as having experienced menopause silently, often without language or support to understand what they were experiencing. Several participants reflected retrospectively on recognising menopausal symptoms in their mothers only later in life.

“We didn’t realize what she was going through. She never spoke about it…We just thought she’s just having severe migraines, but we didn’t realise what they were related to, why they were happening…” (Atifa, 58, Postmenopausal)

In contrast, participants often described increasing openness within their own generation and anticipated that younger women would experience menopause with greater awareness and discussion.

“we’re the sort of newer generation our cultural thoughts are completely different to what our parents were but it’s still that little bit at the moment the new generation obviously are going to be well trained the girls you know they’re going to know and it’s going to be all okay” (Riya, 46, Early Perimenopausal)

These accounts suggest menopause was experienced not solely as an individual biological transition, but within wider relational and cultural expectations that shaped how women managed symptoms, sought support, and understood their changing roles within family life.

#### Healthcare encounters: Dismissal and validation

Participants described healthcare encounters as playing a significant role in shaping how they understood and managed their menopausal symptoms. Many women reflected on experiences of dismissal within healthcare settings, describing consultations in which symptoms were minimised, attributed solely to stress or ageing, or addressed without meaningful discussion or explanation. Several participants described feeling unsupported and uncertain following consultations, reinforcing feelings of frustration and invisibility.

“…it’s just been dismissed, it’s kind of been brushed off. Yeah. Here’s a website, off you pop.” (Meera, 51, Late Perimenopausal)

“I felt like I didn’t have I didn’t feel I had any support from the doctors in the sense that it was literally like, oh, yeah, you know, you’ve gone through it now. And basically, that’s it.” (Fatima, 58, Postmenopausal)

Several women described frustration where emotional symptoms such as anxiety, low mood, or burnout were interpreted primarily through a psychological framework, rather than recognised within the context of menopause. Participants commonly described being offered antidepressants, which some perceived as reflecting limited understanding of the complexity of menopausal experiences.

“Because they don’t understand that…You’re going in with symptoms of anxiety and depression. Then they’re going to allocate back to you. Oh, that’s what you need. Here, have some Prozac. That’s not what we need.” (Bushra, 51, Late Perimenopausal)

“Here’s antidepressants like you go and speak to somebody about kind of your stress and your burnout…we’ve got to become much more aware that menopause is not about missed periods.” (Leila, 45, Postmenopausal)

In contrast, some participants described positive experiences where healthcare professionals and community initiatives provided accessible information and validation. Exposure to menopause education events, culturally relevant discussions, and clearer explanations of symptoms often enabled women to better recognise and contextualise their experiences.

“They were talking about menopause; they had a screen up and they had about 50 symptoms. And I just sort of sat there and I went, I’ve that, I’ve that, I’ve got that. It was like a little pick list.” (Neha, 48, Early Perimenopausal)

“At the community event, we had two doctors who did a role play to help us understand. One was a doctor and one pretended to be a patient, and I could see myself in that role play.” (Aisha, 61, Postmenopausal)

These accounts suggest validating and informative healthcare interactions could enhance women’s understanding of menopause and engagement with support, whereas dismissive encounters often reinforced uncertainty, frustration, and disengagement from care.

### Domain 2: Coping, support, and access to information

#### Self-management and coping strategies

In response to their symptoms, participants described developing a range of self-management and coping strategies to regain a sense of control and emotional stability. Many women reflected on the importance of finding approaches that felt personally meaningful and manageable within the context of their daily lives and responsibilities.

Faith and spirituality were frequently described as important sources of comfort, resilience, and emotional coping. Several participants described religion as providing reassurance and a framework through which to manage distress and uncertainty, particularly where biomedical explanations alone felt insufficient in addressing the emotional dimensions of menopause.

“My religion, so my praying, my Quran, I listen to a lot of Quran, that’s the only thing that really, really helps me…my faith is actually what actually helps me a lot when it comes to tackling the emotional and even…all the certain symptoms apart from the medical ones.” (Riya, 46, Early Perimenopausal)

Alongside spiritual coping, women commonly described adopting lifestyle-based strategies including exercise, dietary changes, natural remedies, and efforts to reduce stress. Participants often framed these approaches as ways of improving both physical and emotional wellbeing, whilst maintaining a sense of independence and routine.

“I think the gym, the gym is a lifesaver…And now it’s just kind of part and parcel of my life.” (Atifa, 58, Postmenopausal)

Several participants also expressed a preference for natural or self-directed approaches over medical interventions, particularly where previous healthcare experiences had felt dismissive or unhelpful.

“I’m generally geared towards more natural medicines and more natural stuff than the artificial doctor, GP, nurse stuff. I’m trying to stay away from that.” (Atifa, 58, Postmenopausal)

For some women, coping strategies were also closely tied to resilience and endurance, with participants describing a need to continue functioning despite ongoing symptoms.

“I am quite resilient and I just get on with it.” (Noor, 53, Late Perimenopausal)

Women frequently framed coping strategies as extending beyond symptom management alone, instead functioning as ways of restoring control, maintaining emotional wellbeing, and continuing everyday functioning within broader cultural and healthcare contexts.

#### Knowledge gaps and informal support networks

Most participants described entering menopause with limited prior knowledge or preparation, frequently citing inadequate education, scarce community resources, and a lack of culturally relevant information as barriers to understanding menopausal changes. Several women reflected that, despite awareness that menopause would eventually occur, they were unprepared for the breadth, duration, and emotional impact of symptoms.

“Like you prepare girls for periods so well. I, you know, I don’t know anyone who was surprised when they got their period. But I do know a lot of women who are surprised by perimenopause.” (Simi, 47, Early Perimenopausal)

Participants commonly described menopause education as largely absent throughout school, healthcare, and community settings, particularly in relation to emotional and psychological symptoms.

“We didn’t even we didn’t learn about it. We didn’t get taught about it. We there was nothing. Nothing about female menopause and how it relates to mental health.” (Leila, 45, Postmenopausal)

Several women also highlighted the limited availability of culturally and linguistically accessible resources, particularly for older generations or women with limited English proficiency.

“I know with our generation English isn’t an issue, but I think maybe for some women, and especially those that come from abroad…make sure that there is support available in other languages yeah which they can maybe identify with …” (Tania, 52, Postmenopausal)

In response to these gaps, many participants described proactively seeking information through informal support networks, including conversations with friends, family members, online resources, and community or faith-based groups. Women frequently described these spaces as more approachable and culturally meaningful environments in which to share experiences, exchange advice, and normalise menopausal symptoms.

#### Male understanding and engagement

Participants consistently described a significant lack of menopause awareness among male relatives, which often affected the level of emotional and practical support available to women during the menopausal transition. Several women reflected that menopause remained poorly understood within family environments, particularly where conversations surrounding women’s health had historically been absent or considered inappropriate.

“So if us women don’t know, then the men haven’t got a bloody clue.” (Leila, 45, Postmenopausal)

Many participants described frustration that emotional and psychological symptoms were often misunderstood or minimised by male partners and relatives, particularly where symptoms were not outwardly visible.

“With men, because men just don’t get it because it’s not a physical illness or physical thing that they can see. They don’t understand the emotional side of it.” (Riya, 46, Early Perimenopausal)

Some women reflected this lack of understanding could contribute to feelings of isolation and insufficient support within relationships.

“You need your family to be supportive, but they can’t be if they don’t know what to look out for or what’s going to happen.” (Meera, 51, Late Perimenopausal)

At the same time, participants described signs of generational and relational change, with some women reporting greater openness and emotional support from husbands, sons, and younger male relatives. Several participants contrasted contemporary family dynamics with earlier generations, where discussions surrounding menstruation and menopause were often hidden from men entirely.

“My mum…she hid it from my father when I was 11, 12 when I had my periods and daddy wasn’t aware…So I think it’s about how you make that a norm…that’s part and parcel of educating the men and the boys as well.” (Atifa, 58, Postmenopausal)

Participants frequently emphasised the importance of involving men in menopause education, describing greater awareness among male relatives as an important facilitator of empathy, communication, and family support during the menopausal transition.

## Discussion

This study contributes to the limited UK qualitative literature exploring menopause experiences among British South Asian women by examining how cultural norms, relational dynamics, and healthcare encounters shape symptom recognition, coping, and help-seeking behaviours. The findings demonstrate that menopause was experienced not solely as a biological transition, but as a socially and culturally mediated process influenced by silence, stigma, intergenerational expectations, and healthcare interactions. While previous research has identified symptom differences and barriers to support among ethnic minority women,^4–6^ the present study extends this literature by illustrating how these factors interact to shape women’s understanding of menopause and engagement with care within a UK South Asian diaspora context.

Participants frequently described uncertainty in recognising and attributing symptoms to menopause, particularly during early perimenopause. Symptoms such as mood disturbance, cognitive changes, sleep disruption, and musculoskeletal pain were often initially interpreted as stress, ageing, or consequences of wider life pressures. These findings align with previous literature describing menopause as an ambiguous and poorly recognised transition, particularly where menopause literacy is limited.^11,13^ Importantly, evidence suggests some South Asian populations may experience menopause earlier than White British populations,^4^ which may further complicate symptom recognition among women in their forties who may not anticipate menopause-related changes. The findings therefore suggest that disparities in menopause care may arise not simply from symptom differences, but from culturally shaped interpretations of those symptoms and delayed recognition of menopause.

Cultural silence and stigma emerged as central influences on women’s experiences and meaning-making processes. Participants frequently described menopause and broader reproductive health issues as topics that were historically hidden within families and communities, resulting in limited intergenerational learning and feelings of isolation. Similar findings have been reported internationally, where menopause within South Asian communities is often framed as a private or inevitable aspect of ageing rather than a health transition requiring support or intervention.^5,13,15,20^ However, the present study extends this literature by demonstrating how these silences continue to shape experiences within a UK context despite increasing public awareness and media discussion surrounding menopause. Access to information alone may therefore be insufficient to reduce inequities where cultural norms continue to influence whether menopause is recognised, discussed, or legitimised.

Participants also described an emerging generational shift towards greater openness about menopause. Women contrasted their experiences with older generations who were perceived to have endured menopause silently and without support. Younger generations, including daughters and younger male relatives, were described as more willing to engage in conversations around women’s health and menopause. These findings support wider literature suggesting that menopause experiences are socially negotiated and shaped by changing cultural narratives and health discourses.^3,9^ Rather than representing a fixed cultural experience, menopause appeared to be situated within evolving intergenerational identities influenced by migration, acculturation, and increased public visibility of menopause in the UK.

Healthcare encounters were particularly influential in shaping women’s perceived control, coping, and willingness to seek support. Many participants described feeling dismissed during consultations, particularly when psychological or emotional symptoms were attributed solely to stress, anxiety, or ageing. These findings align with recent UK qualitative research reporting dissatisfaction with menopause consultations among Black and Asian women, alongside concerns surrounding stereotyping, limited representation, and inequitable access to hormone therapy.^8,21^

These findings are also consistent with recent qualitative findings by Hendriks et al.,^22^ who described delays in recognising menopause, misattribution of psychological symptoms, and the importance of timely, appropriate menopause care in improving women’s experiences. For participants in the current study, dismissive healthcare encounters appeared especially detrimental when occurring alongside pre-existing cultural silences and uncertainty. In contrast, validating interactions characterised by explanation, empathy, and collaborative discussion fostered greater confidence, symptom understanding, and openness toward treatment options. Collectively, these findings reinforce the importance of culturally responsive healthcare, timely recognition of menopausal symptoms, and clinician education that acknowledges both the biological and psychosocial dimensions of menopause.

The present findings also highlight menopause as a relational experience negotiated within family and community systems rather than solely an individual transition. Participants frequently described balancing menopause symptoms alongside caregiving responsibilities, employment demands, and family expectations. Similar relational pressures have been identified in previous UK menopause research, particularly among women from minority ethnic backgrounds managing menopause alongside broader structural and social inequalities.^8,23^ The current study extends this literature by demonstrating how limited understanding from family members, particularly male relatives, contributed to feelings of invisibility, frustration, and emotional isolation. While male understanding remains relatively underexplored within menopause research, participants consistently framed men’s lack of awareness as a significant barrier to support. However, some women also described greater openness among younger male relatives and partners, suggesting educational initiatives involving men may play an important role in reducing stigma and improving family support within collectivist cultural contexts.

Women demonstrated considerable resilience through self-directed and culturally embedded coping strategies. Faith and spirituality were frequently described as important sources of comfort, meaning, and emotional regulation, while exercise, dietary changes, peer discussions, and natural remedies were commonly used to regain a sense of control over symptoms. Rather than functioning as alternatives to medical care, these strategies often complemented women’s broader efforts to manage menopause within contexts where formal support was perceived as limited or inaccessible. Informal peer networks and community discussions were particularly important in normalising symptoms and reducing uncertainty. These findings support previous research emphasising the importance of culturally meaningful coping practices and community-based support systems among ethnic minority women.^10^

### Implication for practice

The findings of this study carry important implications and considerations for healthcare, education, and policy. Within healthcare practice, there is a pressing need for primary care professionals to hold in mind an extended and culturally responsive understanding of menopause management beyond biomedical symptom presentations alone. Participants’ accounts illustrated empathy, validation, and openness to discussing treatment options substantially shaped women’s perceptions of controllability and engagement with care. National guidance has similarly emphasised the need to address disparities in menopause care among ethnically diverse populations and improve culturally informed menopause support within primary care settings.^4,24^ By adopting a culturally nuanced understanding of menopause beyond symptom presentation alone, clinicians may help reduce experiences of dismissal, uncertainty, and inappropriate psychological medicalisation.

Beyond the healthcare setting, the findings of this study highlight community education as a vital avenue for intervention. Participants described significant gaps in menopause literacy alongside limited culturally relevant information, particularly for older generations and women with reduced English proficiency. Existing community reports and culturally tailored intervention literature similarly identify stigma, lack of awareness, and barriers to menopause discussion within South Asian communities.^12,20^ Culturally tailored resources, such as multilingual educational materials, community workshops, and faith-based awareness events, may therefore help respond to current knowledge gaps and improve confidence in recognising and discussing menopausal symptoms. Participants additionally highlighted the pivotal role of informal peer networks in normalising symptoms and exchanging advice. Building upon these existing strengths through structured community initiatives may foster greater shared understanding, reduce stigma, and enhance women’s confidence in seeking medical and social support.

Findings from the present study also emphasised the importance of culturally familiar and trusted environments for menopause education and support. Participants described community centres, women’s groups, mosques, and temples as spaces in which conversations surrounding menopause felt more accessible and less stigmatised. Similar recommendations have emerged within community-based UK menopause initiatives focused on South Asian communities, which emphasise the value of trusted community settings in improving engagement and reducing isolation.^20,21,25^ Community-embedded approaches may therefore provide a more culturally meaningful route to menopause education and support than reliance on formal healthcare environments alone.

An additional key implication related to the inclusion of men within menopause education and support initiatives. Participants consistently described limited understanding of menopause among male relatives, which often contributed to feelings of invisibility, frustration, and reduced emotional support. Emerging evidence suggests that within some South Asian family and community contexts, male relatives may also act as important facilitators or barriers to women’s healthcare engagement and menopause management.^20^ Healthcare professionals working with ethnically diverse populations have reported limited menopause literacy among men may reinforce stigma, minimise women’s symptoms, and contribute to menopause being managed privately within households.^23^ However, participants in the present study also described increasing openness among younger male relatives and partners, suggesting opportunities for more inclusive educational approaches. Family-centred and community-based menopause education involving men, faith leaders, and wider community networks may therefore play an important role in fostering empathy, improving communication within households, and reducing stigma surrounding menopause discussions. Approaching menopause education in a more integrative format may be particularly important within collectivist cultural contexts where family relationships strongly shape women’s emotional wellbeing, support systems, and healthcare engagement.

At the policy level, the findings of this study suggest a need for menopause education and support to be more clearly embedded within public health frameworks, with careful attention to cultural inclusivity. The Women’s Health Strategy for England^26^ identifies menopause and disparities affecting ethnic minority women as key national priorities; however, participants’ experiences suggest gaps remain between policy aspirations and women’s lived experiences of healthcare access and support. National awareness campaigns and menopause strategies must therefore move beyond generalised narratives to more explicitly acknowledge the cultural, relational, and linguistic diversity shaping menopause experiences. Recognising these differences may support the development of more equitable, representative, and culturally responsive menopause care and health promotion initiatives.

### Strengths and limitations

This study adds to the limited UK qualitative literature exploring menopause experiences among British South Asian women and provides insight into how menopause is understood within cultural, relational, and healthcare contexts. The study also highlights emerging generational shifts and the importance of relational support systems, including male engagement, which remain relatively underexplored within menopause research.

Several limitations should also be acknowledged. The sample consisted solely of English-speaking British South Asian women recruited through online and community networks, which may have excluded women with lower English proficiency, reduced digital access, or more marginalised experiences of menopause and healthcare engagement. Participants may therefore have represented women who were comparatively more open to discussing menopause and more engaged with healthcare or community support.

In addition, the sample was predominantly composed of women with university-level education, which may limit the transferability of the findings to British South Asian women whose experiences of menopause literacy, healthcare access, and support may differ. As a result, the findings cannot be assumed to reflect the experiences of all British South Asian women and should instead be understood as exploratory insights into a specific group of women navigating menopause within UK cultural and healthcare contexts.

In addition, participants from different South Asian backgrounds were analysed collectively. While appropriate for the exploratory aims of the study, this may have obscured important intra-community differences relating to religion, migration history, language, and cultural norms. Finally, as with all qualitative research, interpretations were shaped by the researcher’s positionality despite ongoing reflexive engagement throughout the research process.

### Implications for future research

Future research should employ multilingual and participatory approaches to capture a broader and more diverse range of experiences. This would include consideration of women with limited English literacy or reduced access to community networks. Approaches such as the use of interpreters, translated materials, or bilingual researchers may enable more meaningful inclusion of women whose experiences are often underrepresented.

Participatory and co-produced research approaches may be particularly valuable in capturing intra-community diversity and addressing epistemic power imbalances. Emerging openness among younger men suggests that inclusive education may enhance family support and reduce stigma. These require further investigation and consideration in ongoing research. Longitudinal or comparative studies could further explore how understandings of menopause, coping strategies, and help-seeking behaviours evolve across cultural and generational contexts.

### Conclusion

In conclusion, this study provides exploratory insight into how a sample of British South Asian women in the UK understood and navigated menopause within cultural, relational, and healthcare contexts. Findings suggest menopause experiences were shaped not only by biological changes, but also by cultural silence, intergenerational dynamics, healthcare encounters, and broader support systems. The study highlights the importance of culturally responsive menopause care, improved menopause literacy, and inclusive community-based approaches that recognise the diversity of women’s experiences and support needs.

## Supplemental material


Supplemental material - Menopause experiences among British South Asian women: An exploratory qualitative study
Supplemental material for Menopause experiences among British South Asian women: An exploratory qualitative study by Shahara Aziz Ellahi, Vikram Talaulikar, Jessica Wibisono, Aimee Spector in Women’s Health

Supplemental material - Menopause experiences among British South Asian women: An exploratory qualitative study
Supplemental material for Menopause experiences among British South Asian women: An exploratory qualitative study by Shahara Aziz Ellahi, Vikram Talaulikar, Jessica Wibisono, Aimee Spector in Women’s Health

## Data Availability

The qualitative interview data generated and analysed during this study are not publicly available due to sensitive nature and the risk of participant identification.*
