# Supplementary material for: Menopause experiences among British South Asian women: An exploratory qualitative study
Source: Womens Health (Lond). 2026 Aug 11;22:17455057261478338. doi: 10.1177/17455057261478338 (PMC13462505; doi:10.1177/17455057261478338)
Supplement: Supplementary file 1 [file sj-pdf-1-whe-10.1177_17455057261478338.pdf]

Personal Experience with Menopause:

- Can you describe any menopause symptoms you are currently experiencing or have experienced in the past?
- How have these symptoms affected your daily life and well-being?
- How has your experience of menopause had a wider impact, if any, with your family, friends, and community?

Emotional and Mental Health:

- How has menopause affected your emotional well-being? Do you experience any anxiety, depression, or stress related to menopause?
- What strategies do you use to maintain your emotional health during this transition?
- Are there specific cultural or societal factors that make it difficult for you to seek mental health support for your symptoms of menopause?

Cultural Context and Support:

- How has your cultural background influenced your understanding of menopause and seeking support?
- Are there specific cultural or societal factors that make it difficult for you to seek support for your menopause symptoms?
- Are there any cultural practices or remedies that are commonly used for managing menopause symptoms in your community?

Support Systems:

- What kind of support, if any, have you sought for managing your menopause symptoms? If so, how was this experience? This may include family, friends, community hubs.
- What are your recommendations on how support for menopause could be improved for women in your community?

Raising Awareness:

- What can be done to increase awareness about menopause in the South Asian community?
- How should information about menopause be communicated to women in your community?

Final Thoughts:

- Is there anything else you would like to share about your experience with menopause or your thoughts on how it is managed in your community?
