# Supplementary material for: Menopause experiences among British South Asian women: An exploratory qualitative study
Source: Womens Health (Lond). 2026 Aug 11;22:17455057261478338. doi: 10.1177/17455057261478338 (PMC13462505; doi:10.1177/17455057261478338)
Supplement: Supplementary file 2 [file sj-pdf-2-whe-10.1177_17455057261478338.pdf]

Domain	Sub Theme	Quotes
**Experiences and understanding of menopause**	**Self-management and coping strategies**	"It's an Asian thing; we don't talk about it…I didn't know anything about it and just got on with it." **(Aisha, 61, Postmenopausal)**<br><br>"Topics about menopause, it's really kept secret. It's not really announced. It's not really publicized." **(Priya, 43, Early Perimenopausal)**<br><br>"I've never ever sat in a space with middle-aged women, women who are older than me even, who have ever really spoke about it, it's this shameful thing." **(Fatima, 58, Postmenopausal)**<br><br>"I think in Asian culture you don't talk about this kind of thing. And that's quite hard because you don't know who you talk to." **(Zara, 49, Postmenopausal)**<br><br>"Some women out there who don't even know that they're going through it but they're dealing with these symptoms and haven't got a clue …you have these emotions, and you have these feelings that you're not allowed to, you've got to suppress them because you're not allowed to talk about them." **(Riya, 46, Early Perimenopausal)**<br><br>"It's just so uncomfortable to talk about, you feel like your doing something wrong" **(Saba, 58, Late Perimenopausal)**
**Experiences and understanding of menopause**	**Physical and emotional disruption**	"Tinnitus, sweats, broken sleep, mood swings, frozen shoulder, cloudy brain, difficult to recall, bloating and gas, itchy skin," **(Neha, 48, Early Perimenopausal)**<br><br>"Brain fog, clumsy, the pains, the mood swings. My breath's hurting, that started all of a sudden." **(Riya, 46, Early Perimenopausal)**<br><br>"There were the sweats, discomfort during the night, difficulty sleeping, this hotness in your system, but also this loss of sort of this low mood, depression, anxiety" **(Priya, 43, Early Perimenopausal)**

		"Hot flushes that was. That was one of them. I never. I've never been a hot person. I'm always a cold person. So like getting hot, hot flushes, night sweats. Vaginal dryness... like aches and pains, joint pain." **(Leila, 45, Postmenopausal)**<br><br>"I kept thinking, is this the menopause or is it just the stress of my divorce? Everything was happening at once, I couldn't separate it. Because I think even now sometimes, if I'm honest, you know, I do question, would it have happened if I'd not been going through the menopause? You know, how much of the menopause was actually linked to the things that might have been getting to me more than they were getting to me before." **(Tania, 52, Postmenopausal)**<br><br>"You feel like a different person because you you've dealt with, you being you as a person throughout your life and then all of a sudden you feel like a different person, like something's being taken away from you. So I feel like I'm not the person that I used to be that I'm so used to being." **(Simi, 47, Early Perimenopausal)**<br><br>"It's a complete it's a complete shift in identity like you become, you're a version of yourself, but you're a much more blunted, duller version of yourself, is the is the best way I can describe it. So to everyone else, you look like you and they can't understand why you're not being you, but they don't see what's what's going on inside." **(Leila, 45, Postmenopausal)**<br><br>"And I cry a lot as well. I just feel sad and I cry and I think of the worst stuff that's gonna happen to me...because I live by myself now, because my daughter got married a couple of months ago in October. So I live by myself and I think I have more time to think about sad stuff than good stuff." **(Ariana, 57, Postmenopausal)**<br><br>"I was so nervous. I was driving home...and I just stopped the car on the motorway and I was like, I can't, I can't drive. I can't do anymore...I totally lost my confidence" **(Bushra, 51, Postmenopausal)**<br><br>"You feel like a different person...like something's being taken away from you. So I feel

		like I'm not the person that I used to be that I'm so used to being." **(Simi, 47, Early Perimenopausal)**
**Experiences and understanding of menopause**	**Roles, relationships, and generational change**	"if my nan was here, she'd be saying, my goodness gracious, like we didn't have to work. Our sole concern was buy the food, cook the food, feed the food...You guys are going out and working. You've got your children who are doing various different things. There's so many strands to our like modern life and that it's just different." **(Neha, 48, Early Perimenopausal)**<br><br>"I really struggled at home to do little things, things I got very overwhelmed with, like, you know, even the very basic things. Like housework and stuff like that's where things went down, you know, and my mood, I suppose, and people living with me, poor them, you know, getting annoyed with them more than I normally would about stuff." **(Tania, 52, Postmenopausal)**<br><br>"I'm going off to do this you know for myself from mental health or whatever. It's like, well, what about your kids? What about your husband? What about, you know, your responsibilities to everybody else?" **(Tania, 52, Postmenopausal)**<br><br>"I feel a whole lot of reluctance doing some certain things because of my mood swings, sleep disturbance, getting off rest and it's really a problem to me having to pay a good and close attention to my family. So it's really affecting my family especially." **(Priya, 43, Early Perimenopausal)**<br><br>"...you're kind of just going through life on your own, expected to get on with it and cope as best as you can." **(Atifa, 58, Postmenopausal)**<br><br>" So how do you this on your own? How? How can you get through these dark days on your own?" **(Bushra, 51, Late Perimenopausal)**

		"I’ve always been open to my mum anyway. But I would say to her things like, you know, my pee is really heavy. she’ll say oh you know you’re coming to that age and it’s part of the process...it’s been nice to have someone to say it’s normal you know it’s fine or you need to go and see a doctor because that doesn’t sound quite right so it’s it’s been quite helpful" **(Meera, 51, Late Perimenopausal)**<br><br>"I’ve told my husband I’m going to start snapping at you to just deal with it. So he’s being very good. He’s just changed as a person. So Alhumdulillah, I can’t. He’s just accommodating all of my issues." **(Noor, 53, Late Perimenopausal)**<br><br>"I have good boys, they’re very supportive, they’re very active, they’re well aware of mum’s ailments and the fact that she’s going through a change. I can even discuss it with them, I’m very fortunate." **(Noor, 53, Late Perimenopausal)**<br><br>"I do get support from my my sisters. We do discuss a lot of things. then one or two or three of them are going through menopause. So we have kind of the similar symptoms, which kind of helps. That does help." **(Atifa, 58, Postmenopausal)**<br><br>"Asian women don’t have hormones or menopause, do you know what mean? Basically your periods stop and that’s it. But all the symptoms and everything that’s attached to menopause, it’s kind of like your elders, they kind of see it as well. Well, I went, you know, my period stopped, I was fine, you know, pull yourself together sort of thing, you know." **(Tania, 52, Postmenopausal)**<br><br>"Menopause wasn’t recognized when my parents, my mom was around because it was just like, oh, mom’s mom’s got issues, but we didn’t recognize it. It menopause when she, what she was going through. So it’s the same. It does affect all your relationships." **(Riya, 46, Early Perimenopausal)**

		"we didn't realize what she was going through. She never spoke about it. Yet she had regular migraines...now I understand, you know all everything she went through that unfortunately She didn't talk about it and we couldn't help her in that area. We just thought she's just having severe migraines, but we didn't realise what they were related to, why they were happening..." **(Atifa, 58, Postmenopausal)**<br><br>"we're the sort of newer generation our cultural thoughts are completely different to what our parents were but it's still that little bit at the moment the new generation obviously are going to be well trained the girls you know they're going to know and it's going to be all okay" **(Riya, 46, Early Perimenopausal)**<br><br>"There's a couple of sisters that have done a lot of talks about menopause. My younger sister keeps giving me stuff…it's very, I'm very fortunate that I'm of that generation, that there is so much more awareness...so I think really I'm of that generation where, you know, I know where to go." **(Noor, 53, Late Perimenopausal)**<br><br>"I think because because we're born here and brought up here, I think we've got the colour and we've got the we've got the culture. But we've been brought up here, haven't we? So it's kind of different, isn't it? We we don't necessarily fit in that box or that box. We kind of like a bit of both." **(Tania, 52, Postmenopausal)**
**Experiences and understanding of menopause**	**Healthcare encounters: dismissal and validation**	"it's kind of like, been like you said, you've captured the word perfect perfectly, and that it's just been dismissed, it's kind of been brushed off. Yeah. Here's a website, off you pop." **(Meera, 51, Late Perimenopausal)**<br><br>"I felt like I didn't have I didn't feel I had any support from the doctors in the sense that it was literally like, oh, yeah, you know, you've gone through it now. And basically, that's it."

		**(Fatima, 58, Postmenopausal)**<br><br>" Here's antidepressants like you go and speak to somebody about kind of your stress and your burnout. And I'm like. How old are you? OK, you're 43. OK, what are your hormones doing? It's, you know, we've got to become much more aware that menopause is not about missed periods, like that's what your GPS gonna say. No, no, you're not perimenopausal. You've still got regular periods. That's the last ******* thing to go for." **(Leila, 45, Postmenopausal)**<br><br>"because they don't understand that. There's nothing, not doing it to be sort of mean or anything like that. They don't understand. You're going in with symptoms of anxiety and depression. Then they're going to allocate back to you. Oh, that's what you need. Here, have some Prozac. That's not what we need." **(Bushra, 51, Late Perimenopausal)**<br><br>"It came to sort of World Menopause Day and Menopause Medical Centre...They put on a South Asian women's health awareness event...they had two gynaecologists on a panel. And they were talking and when they were talking about menopause, they had a screen up and they had about 50 symptoms. And I just sort of sat there and I went, I've that, I've that, I've got that. It was like a little pick list." **(Neha, 48, Early Perimenopausal)**<br><br>"At the community event, we had two doctors who did a role play to help us understand. One was a doctor and one pretended to be a patient, and I could see myself in that role play." **(Aisha, 61, Postmenopausal)**<br><br>"The good thing is they even send you links now. Which is brilliant. You send a link, you find all these numbers, the way the council are setting churches and community centre, which is brilliant." **(Ariana, 57, Postmenopausal)**
**Coping, support, and access to information**	**Self-management and coping**	"My religion, so my praying, my Quran, I listen to a lot of Quran, that's the only thing that really, really helps me. And praying, those sort of things that really, and my faith is actually what actually helps me a lot when it comes to tackling the emotional and even, yeah, all the

	**strategies**	all the certain symptoms apart from the medical ones, yeah." **(Riya, 46, Early Perimenopausal)**<br><br>"I think the gym, the gym is a lifesaver... it's taken me a couple of years to kind of...get to that point...And now it's just kind of part and parcel of my life. And it's a very important factor that slots in" **(Atifa, 58, Postmenopausal)**<br><br>"I'm generally geared towards more natural medicines and more natural stuff than the artificial doctor, GP, nurse stuff. I'm trying to stay away from that." **(Atifa, 58, Postmenopausal)**<br><br>"I am quite resilient and I just get on with it" **(Noor, 53, Late Perimenopausal)**
**Coping, support, and access to information**	**Knowledge gaps and informal support networks**	"Like you prepare girls for periods so well. I, you know, I don't know anyone who was surprised when they got their period. But I do know a lot of women who are surprised by perimenopause." **(Simi, 47, Early Perimenopausal)**<br><br>"We didn't even we didn't learn about it. We didn't get taught about it. We there was nothing. Nothing about female. Female menopause and how it relates to mental health." **(Leila, 45, Postmenopausal)**<br><br>"so I knew it was going to happen, but I wasn't prepared for it. I wasn't prepared for perimenopause. I wasn't prepared for all those symptoms and what it entails and how long it lasts." **(Simi, 47, Early Perimenopausal)**<br><br>"I know with our generation English isn't an issue, but I think maybe for some women, and especially those that come from abroad...make sure that there is you know support available in other languages yeah which they can maybe identify with a little bit more even just terms they can identify with." **(Tania, 52, Postmenopausal)**

**Coping, support, and access to information**	**Male understanding and engagement**	"So if us women don't know, then the men haven't got a bloody clue." **(Leila, 45, Postmenopausal)**<br><br>"With men, because men just don't get it because it's not a physical illness or physical thing that they can see. They don't understand the emotional side of it." **(Riya, 46, Early Perimenopausal)**<br><br>"You need your family to be supportive, but they can't be if they don't know what to look out for or what's going to happen." **(Meera, 51, Late Perimenopausal)**<br><br>"If women don't understand what it is and aren't told about it, then I don't see how the men can understand it. And therefore I don't think that support is there for the women." **(Meera, 51, Late Perimenopausal)**<br><br>"my husband thinks I've gone mad, but because the thing is very hard to explain menopause to men. it is a little bit difficult to explain to because he doesn't get it." **(Riya, 46, Early Perimenopausal)**<br><br>"They almost think, oh, it's no, it's just fine. It's in your head. It's in your head. Like you can make it. It's all psychological." **(Riya, 46, Early Perimenopausal)**<br><br>"I know these issues in our community especially have been very hush-hush when we had our periods years ago, you know, My mum...She hid it from my father when I was, I remember when I was 11, 12 when I had my periods and daddy wasn't aware, you know, daddy could not be told...And yet when my husband was alive, you know, he the girls would faint when they were on the period. He would know about it. He would help them. He would support them...So I think it's about how you make that a norm...that's part and parcel of educating the men and the boys as well." **(Atifa, 58, Postmenopausal)**<br><br>"My husband understands that I've gone through this whole many changes and things. So he's become a lot more patient with me compared to how it would normally be." **(Riya, 46, Early Perimenopausal)**

		"it's really important to engage the men, you know, in this transition...If we are able to educate the men, husbands and fathers, uncles, know, about menopause, you know, it could go a long way to foster empathy, to have a better support for women, Asian women, and to have a good dialogue within the family and to make the journey smoother and better." **(Priya, 43, Early Perimenopausal)**
